# Three tips for creating an online course for nontraditional student populations

**DOI:** 10.1002/ece3.7841

**Published:** 2021-06-29

**Authors:** M. Rebecca Forman, John A. Chapman

**Affiliations:** ^1^ Erosion and Stormwater Management Certification Program Department of Bioproducts and Biosystems Engineering University of Minnesota Twin Cities Saint Paul MN USA

**Keywords:** adult education, distance learning, non‐traditional students, remote instruction

## Abstract

In spring 2020, the University of Minnesota Erosion and Stormwater Management Certification Program temporarily ceased in‐person workshops due to the spread of COVID‐19. Twenty workshops were canceled, and the 1,233 attendees (all adult learners) were moved into asynchronous online course sections. These online workshops were the first remote courses that many of the attendees had ever attempted. Here, we provide tips for successfully creating online classes for nontraditional student populations.

## Graphical abstract

Due to the spread of COVID‐19 in the United States, the Erosion and Stormwater Management Program, a certification program for adult professionals, was required to migrate in‐person courses to an online format in Spring 2020. In this paper, we provide tips for successfully creating online classes for non‐traditional student populations.

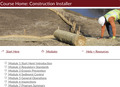

## 1 INTRODUCTION

In March 2020, the novel coronavirus 2019 (COVID‐19) pandemic suddenly and dramatically changed nearly every aspect of life throughout the world. In order to slow the spread of the virus, mass gatherings were prohibited and businesses, restaurants, and schools abruptly closed (World Health Organization, 2020). In‐person learning ceased at the University of Minnesota; therefore, the Erosion and Stormwater Management Certification Program at the University of Minnesota was required to discontinue in‐person classes and move to an online environment. The rapid movement of education to an online format presented challenges to adult learners, many of whom were required to quickly learn new technologies.

In this paper, we provide a brief background on the context of our educational program and, based on our experiences and the feedback we received from our course attendees, we offer three tips for the transition from in‐person professional training to the online environment for a diverse group of adult learners.

## 2 THE EROSION AND STORMWATER MANAGEMENT CERTIFICATION PROGRAM

The Erosion and Stormwater Management Certification Program (hereafter referred to as the Erosion Program, www.erosion.umn.edu) is housed within the Department of Bioproducts and Biosystems Engineering in the College of Food, Agriculture, and Natural Resource Sciences at the University of Minnesota. The Erosion Program was created in 2002 in partnership with the Minnesota Pollution Control Agency (MPCA) and the Minnesota Department of Transportation (MnDOT) to provide construction stormwater certification to MnDOT employees and contractors. The Erosion Program has grown substantially throughout the years and now provides certification and training to a wide audience on numerous erosion prevention and sediment control topics. Between July 2018 and June 2019 (the year prior to the implementation of COVID‐19 restrictions), the Erosion Program offered 16 different course titles, conducted 99 workshops, and provided training to 4,011 adult professionals.

In Minnesota, the MPCA issues Stormwater General Permits to construction projects in accordance with the Clean Water Act. One requirement of the permit is that certain construction personnel receive training commensurate with their job responsibilities. The Erosion Program offers six construction stormwater certification courses that enroll the bulk of our attendees. Three of the courses are initial certification courses: a 2‐day construction site management course designed for construction site supervisors, environmental consultants, and government agency staff; a 1‐day erosion prevention and sediment control product installation course designed primarily for construction laborers (who install the products); and a two‐day construction stormwater plan preparation course that is designed for engineers and scientists. Upon completion of a written examination, the attendee is certified for a period of three years. In order to maintain the certification, the attendee must return for a course‐specific one‐day refresher training every three years. Erosion Program staff have historically traveled throughout the State of Minnesota to offer these one‐ and two‐day courses to local residents. Course materials have been delivered through PowerPoint lectures as well as large‐ and small‐group exercises. The Erosion Program staff have made a concerted effort throughout the past decade to lecture less and to provide more active learning opportunities for the attendees.

The student population for these courses is distinct from that of most university classes. In addition to a wide variety of ages in each course (from 18 to 70+), there is significant diversity in attendees’ education levels. While the stormwater plan preparation course (and its recertification) is typically attended by those with four‐year degrees, this course usually comprises under 20% of the Erosion Program registrants. The bulk of the registrants are individuals who spend their days on construction sites, many of whom have been educated in the trades, but do not hold 4‐year degrees.

Previous studies have examined why adult learners choose to return to higher education (e.g., Dotta et al., 2020) as well as why nontraditional students choose to enroll in online rather than in‐person learning (e.g., Dos Santos, 2020). Furthermore, it has been well documented that nontraditional students drop out of online classes at a higher rate compared to traditional students (Afzal, 2020; Carr, 2000). The majority of these studies examine nontraditional students in college courses. However, not all online education for adult learners is a college course. Numerous professions require certifications or other trainings that must be maintained in order to continue practice (e.g., Occupational Safety and Health Administration (OSHA) trainings, and continuing education). When in‐person training was not available due to COVID, these learners were required to meet their training obligations through online learning—many for the first time. Consistent with findings from Deschacht and Goeman (2015), a large percentage of the Erosion Program attendees self‐identified as new to online learning. Additionally, numerous attendees described themselves as lacking basic computer skills. Online learning requires a good grasp of Internet‐related actions, and for those not comfortable with these actions, online education is challenging (Kuo et al., 2014). Based on our experience, we offer the following tips to instructors moving in‐person classes to an online format.

## 3 TIP #1: KEEP THE TECHNOLOGY PART OF YOUR COURSE SIMPLE

With nontraditional adult learners, we have found it best to keep a relatively simple, low‐tech format. We chose to use short videos, still photographs, and hyperlinked documents as the backbone of our courses. Some technology options that can aid in learning are described below. We feel it is important to weigh the benefits of each additional technology with the potential frustrations and barriers it may pose to our students. Because of the sudden nature of the switch to asynchronous online learning in spring 2020, and the additional stresses many people were facing (including lack of childcare and financial stressors), we opted to eschew using multiple technologies for this iteration of the courses. We feel that with proper time to prepare, and with the proper audience, one or two additional technologies could potentially be added, but we caution against adding too many technology platforms.

**FIGURE 1:**
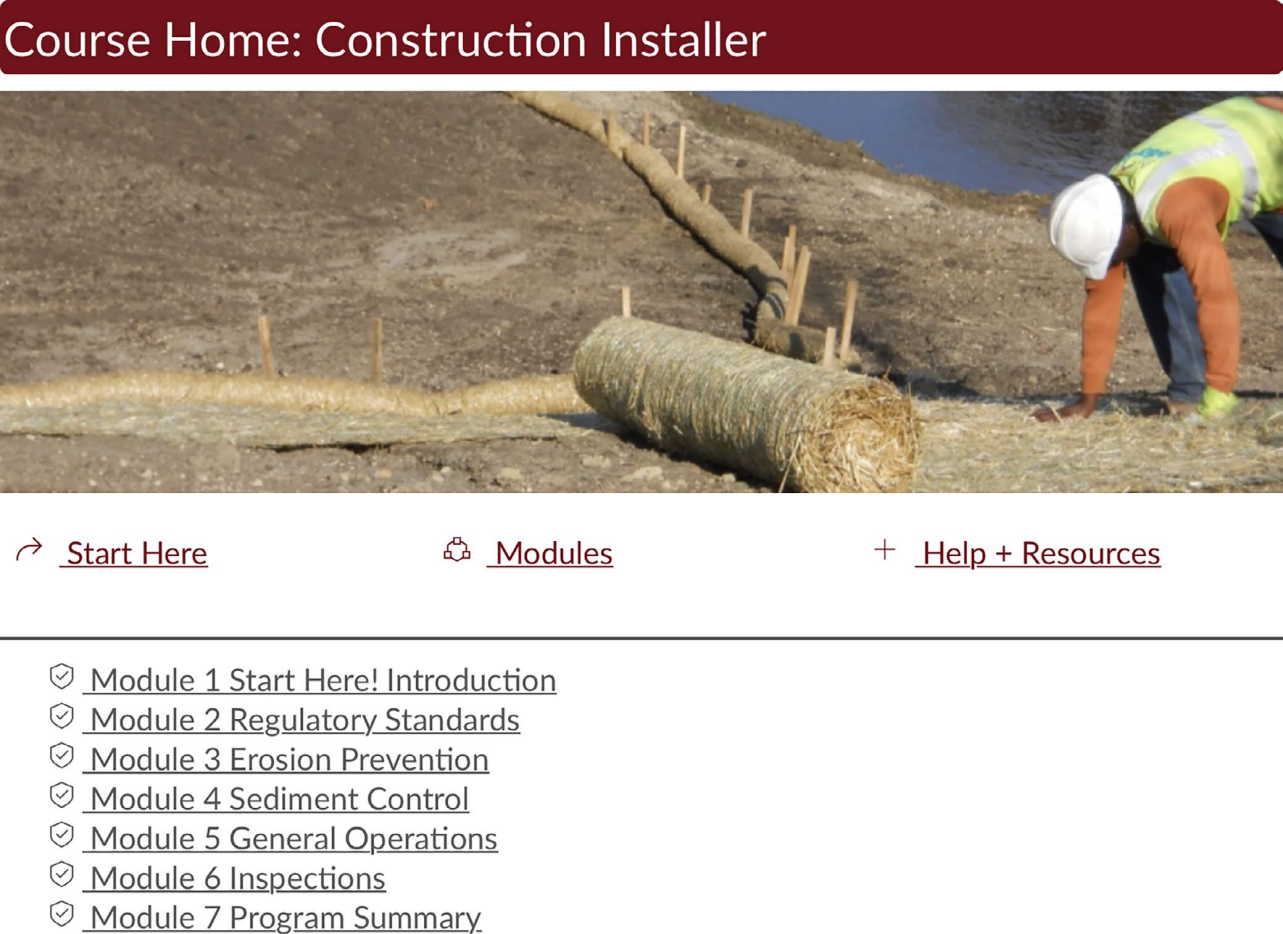
Landing page for one of the Erosion Program online courses

The University of Minnesota uses *Canvas* (https://www.canvas.net) as their learning management system. The Erosion Program created a Canvas course for each of the course titles we offer, and we organized the course content into *modules* within the Canvas framework (see Figure 1 as an example). For each module, we chose to record short (under 12 min) videos using the free Screencast‐O‐Matic (https://screencast‐o‐matic.com) software. If a topic required more than 12 min of content, we broke the topic into multiple videos. These short videos were referenced multiple times in our course evaluations as one of the best parts of the class (especially among those who were self‐described as among our least technologically savvy students). We chose to keep the videos short because we knew from our experiences taking classes that attendee attention wanes when videos get too long. Also, because many of the attendees were working full‐time and taking the course when they had spare moments, and because some of our attendees were also home‐schooling their children while completing the course, these short videos allowed the attendee to complete “chunks” of material in a relatively short amount of time. The videos were captioned, so they are more accessible and provide students with another way to engage with the material. In the future, we would also like to provide captions in Spanish (and potentially additional languages) to aid those students who are more comfortable learning in another language.

Another software option to create videos is VoiceThread (https://voicethread.com). A benefit of the software is that the instructor can narrate each slide individually, and thus, if the instructor wishes to change something about the narration, it is easy to re‐record a single slide. Another benefit of VoiceThread is that attendees can comment on a presentation by inserting a text or audio clip at any point in the VoiceThread narration. This can be a great way to engage students with course discussions. However, with the large number of attendees in each of our courses, as well as the increased time to narrate each slide individually, we chose not to use this software in our initial online offering.

In order to reinforce the video content, attendees were then provided a photograph and were asked to comment on a specific aspect of it (e.g., “What do you think of the installation of this product?”, or “Do you think the site manager chose the correct product to install?”). Canvas provides a “discussion question” format where the instructor can post a photograph (or video or text) and the attendee is required to type his/her contribution to the discussion into a text box. Once the attendee has contributed to the discussion, previously provided discussion contributions from the other attendees are revealed. These discussion questions afforded the attendees a chance for peer‐to‐peer learning in the asynchronous environment. As all attendees were required to post comments using their real names (they were unable to create a username), we had very few problems with attendees not putting forth effort in their answers. Course instructors did moderate the comments to ensure that the visible comments were high quality. We received positive feedback on the “discussion question” format. One attendee wrote:

I was just going to let you know that since I was social distancing at home, I took the Erosion Control Supervisor Class last week. I wanted to let you know you guys put together a good and interesting program… Thanks for making this class very informative and interesting. I thought your review questions after the modules were good and initiated more of a response than a normal class atmosphere. (Well at least the other states programs I have been in class in person).

A second attendee stated:

Enjoying the material and training. It is a great online class. … Only thing missing is class interactions. But the reply with an answer to the photo examples is a good way to "force" everyone to participate. (I have a training background in my "past life" so know I am critiquing as I complete the training.)

Some instructors may find that it can be helpful to “meet” the class attendees of a course in order to increase class engagement. A free app that may be used for this purpose is FlipGrid (https://info.flipgrid.com). In order to keep the class relatively low‐tech, we did not use FlipGrid in our course this past spring. However, we plan to use the app for informal introductions and minipresentations in select future courses (e.g., courses that are primarily attended by engineers and scientists).

We knew that many students were missing out on the ability to ask questions and hear answers in real time. Therefore, we chose to use the ubiquitous Zoom software (https://zoom.us) to hold several question and answer sessions. However, we heard from several attendees that they were unable to attend the Zoom sessions because their employer did not allow that specific software to be run on work computers. Others did not have access to broadband Internet and were unable to use Zoom without the software freezing. Many households (including the authors’) found that even with broadband Internet, when multiple household members had concurrent Zoom meetings, the video and audio quality was poor for everyone in the household. Our solution to this problem was to ask those who were unable to use Zoom to send us any questions they had via email prior to the Q&A session. In addition to providing the attendee with the answer at that time, we also answered their question during the Zoom session in case others in the meeting had a similar question. All Zoom sessions were recorded so those who could not use the software, or who were unavailable during the time of the recording, could watch the recording of the meeting.

## 4 TIP #2: BE FLEXIBLE TO ENSURE PARTICIPANT SUCCESS

We cannot emphasize enough the suggestion to remain flexible in how you run your course. As mentioned, many of the students who took our course were also working full‐time. It is not uncommon in the construction industry that employers expect online courses to be completed when it is raining and therefore construction won't be happening. Sometimes there is not a rainy day between the course assignment and the course due date. Sometimes life (parenting, taking care of sick relatives, etc.) gets in the way of completing classes in a timely manner. We found several ways to be flexible to ensure that as many participants as possible were able to successfully complete the course that did not add undue burden to the instructor's workload.

The Erosion Program was flexible with the methods in which students were required to turn in materials. Two of our courses required attendees to complete and turn in a document. While it would have been easiest for the Erosion Program staff to require all attendees to upload a pdf of the document, we set up the Canvas assignments to allow for multiple formats (docx, pdf, pasted text, or a photo image of printed pages). Our goal was to be as flexible as possible to ensure that the attendees were able to complete the assignment and understand the learning objectives. While these types of format changes may be simple for those who use computers frequently, this was not the case for some of our students. We felt that the little bit of extra effort for Erosion Program personnel was worth not having this technology barrier and frustration for our participants.

As noted in Tip #1, some of the attendees did not have access to broadband Internet. Additionally, numerous attendees were only provided with company phones and thus had no computer on which to work. Another way the Erosion Program was flexible was to offer a “distance learning” course to those attendees. We mailed those students the in‐class workbook (that is not used in the online class) along with several worksheets that could be used in lieu of the videos. To replicate the photograph component of the course, we printed photographs onto 8.5″ × 11″ paper and provided space for the attendee to write comments about each photograph. Obviously, attendees who participated in the distance learning course were unable to see other students’ comments about the same photograph, but when multiple individuals from the same company were taking a course in this format (which occurred), they were able to discuss their findings among themselves.

Many attendees had high stress levels this past spring; when stressed, many people easily forget details (such as due dates). Each June, the Erosion Program prints laminated wallet certification cards for all course attendees for the previous academic year. In order to meet this deadline, we needed all courses completed and scores into our administrative assistant by May 20th. To meet this goal, we set a course completion date of May 1st, and we communicated this date to attendees when they were enrolled in the online class. Approximately 2 weeks prior to May 1st, Erosion Program staff sent an email to all attendees who had not yet completed the course to remind them of the due date and inquire whether anyone needed additional time to complete the course. We repeated this email one week prior to May 1st and then 3 days prior to May 1st. As the date got closer to May 1st, more attendees wrote and asked for an extension, and since we had built that into the course calendar, we were able to grant extensions. After the May 1st deadline had passed, we again contacted attendees who had not completed the course and asked what was needed for them to complete the course. While this level of communication to get participants to complete a course may seem excessive, we found repeated communications and building extra time into our schedule to be effective in assisting with participant completion. Course completion rates varied from 86% to 100% among the seven online classes that we offered, with an average completion rate of 92%. While this rate is lower than the typical in‐person completion rate, we were pleased that everyone who indicated that he/she wished to complete the course was able to complete it by our May 20th date.

Finally, we were flexible with the *when* and *how* we were able to meet with/answer attendee questions. We FaceTimed, we Zoomed, we texted, and we gave out our personal cell phone numbers. We chatted with attendees during business hours, in the early morning and late evening, and on weekends. When communicating one‐on‐one with attendees, our goal was to communicate in whatever format was most convenient for the attendee. However, one aspect of communication that will change in the future is that instructors will be advised to consider using a virtual phone number (such as Google Voice (https://voice.google.com )) or to block his/her phone number (e.g., by dialing *67 prior to the phone number) to allow for instructors to set reasonable limits to phone contact frequency and hours.

We received many emails thanking us for our flexibility with the courses. The following communication is an example of what some of our students were dealing with, and why flexibility was critical. The quote was lightly edited for clarity.

I will be down to the wire Friday [with completing the course]. My days are not my own and I just received an invite for a Sitework Kick Off [on a 100+ acre site] tomorrow morning with the city. If I need to attend (not call in), the drive and meeting will burn up a large portion of my day. My week has been filled with COVID meetings and my usual day‐to‐day grind from overseeing a number of projects throughout the country. If uninterrupted, I should get through the coursework tonight. I’ll update you tomorrow if I anticipate problems concluding this by end of day.

## 5 TIP #3: PROVIDE AND SOLICIT REGULAR FEEDBACK

One of the challenges of an asynchronous class is giving and receiving meaningful feedback. However, the more feedback that is provided to students, the more engaged we found the students to become. We aimed to provide and solicit feedback multiple times throughout the course.

One of the ways in which we provided feedback was by reviewing the attendee‐provided responses to the photograph discussion questions. When warranted, instructors asked probing questions or provided comments to responses. While this may be fairly easy to accomplish in a small class, with 1,233 registered online attendees and multiple discussion questions per course, we found it difficult to keep up. In the future, we plan to provide a “correct answer” slide after the discussion question; attendees would then be asked to compare the answer he/she provided to the correct answer slide in order to encourage reflection and ensure understanding of the learning objective.

When items were turned in, we frequently checked them over, but, since they were not graded, we did not provide attendees with feedback. One of the comments we received was that attendees wanted to know how they did on assignments they turned in. They did not view the assignment as a “check the box,” but rather wanted to ensure that they had mastered the material. In the future, we will either convert the assignment to a format that can be autograded, or ensure instructors respond to each attendee with individualized feedback.

The most common way that we sought feedback was to individually message students to find out how they were doing and what questions they had. Canvas allows the instructor to send out bulk messages that are individually generated to each attendee. This feature provides a sense of direct communication between the instructor and each attendee. We found that without these messages, attendees would rarely send us questions. However, once we opened the lines of communication, attendees began to respond to our messages. We recommend checking in with attendees regularly to solicit any questions that they might have.

In addition to email inquiries, we also provided a short end‐of‐course survey. While the number of surveys that were returned was very small (*n* = 45 plus approximately ten unsolicited feedback emails prior to the survey), attendee evaluations and unsolicited feedback indicated that our classes were informative, interesting, and well done. We asked attendees what they liked best about the class (typical answers included the videos and being able to do the course on the attendee's own timeline), what could be improved (we received numerous comments on the difficulty in getting into the Canvas platform), and what the attendee would recommend to improve the course. This final question provided us with some of the most helpful ideas about ways to improve the class. This year we received feedback that it would be helpful to provide all of the required downloads at the beginning of the course in addition to throughout the course. That way if the attendee chose to print the materials, all of the provided guidance documents would be easy to locate.

## 6 CONCLUSION

Many people envision either K‐12 or colleges/universities when they think about entities that were required to migrate educational content online during spring 2020. However, community colleges, extension educators, and trainers who provide courses that are required due to local, state, and/or federal requirements (such as the courses the Erosion Program offers) were also required to make this shift to an online environment. In a review of literature, Afzal (2020) identified three factors that were repeatedly associated with the retention of students in online course: 1. the level of engagement with materials and peers, 2. the student's self‐regulatory abilities, and 3. the amount and quality of teacher feedback given. We think these factors similarly apply to courses for nontraditional adult learners, but additional attention must be paid to added technology barriers and potentially more complicated out‐of‐class family and work responsibilities. Similar to the findings of Kaiper‐Marquez et al. (2020), who found that adapting a family literacy program from an in‐person to a remote environment, the move to online education significantly increased instructor preparation time, we found that the online course format required additional time from our instructors. Supervisors should be aware of these additional time demands when considering a transition to online formats. While there are additional challenges in creating and facilitating online courses that serve the diverse populations of students that take our courses, we feel that an offering of a variety of course formats (online, in‐person, potential hybrid offerings) will be of continued value in postpandemic times. We hope that the tips provided will be helpful to others who work within the continuing education sector as they decide if online instruction will be beneficial to their students.

## CONFLICT OF INTEREST

None declared.

## AUTHOR CONTRIBUTION

**M. Rebecca Forman:** Conceptualization (equal); Writing‐original draft (lead); Writing‐review & editing (lead). **John A. Chapman:** Conceptualization (equal); Writing‐original draft (supporting); Writing‐review & editing (supporting).

## Data Availability

The contents of this paper are reflections of the experience of the authors during the instructional period described. Data supporting the findings of this study are available within the paper.
